# Associations between short-term exposure to fine particulate matter with ischemic stroke mortality and the role of green space: a time-series study in Zibo, China

**DOI:** 10.7189/jogh.15.04068

**Published:** 2025-03-21

**Authors:** Sihao Song, Chuanlong Cheng, Ying Liu, Yuqi Duan, Hui Zuo, Rui Xi, Zhisong Ni, Kemeng Liang, Shufen Li, Feng Cui, Xiujun Li

**Affiliations:** 1 Department of Biostatistics, School of Public Health, Cheeloo College of Medicine, Shandong University, Jinan, Shandong, China; 2 Ma'anshan Center for Disease Control and Prevention, Ma'anshan, Anhui, China; 3 Zibo Center for Disease Control and Prevention, Zibo, Shandong, China

## Abstract

**Background:**

Previous studies on associations between short-term exposure to fine particulate matter (PM_2.5_) and ischemic stroke (IS) mortality reported inconclusive results. Additionally, whether and how PM_2.5_ and green space interact to precipitate IS deaths remains unclear. We aimed to examine the impacts of short-term exposure to PM_2.5_ on IS mortality and the role of green space in the association.

**Methods:**

We collected data on daily IS deaths, daily PM_2.5_ concentrations, and monthly normalized difference vegetation index (NDVI) in Zibo City from 2015 to 2019. Generalised additive models were adopted to investigate the short-term impacts of PM_2.5_ on IS mortality, and subgroup analyses were used to examine effect modification by population characteristics. Stratified analyses by green space levels and joint effect model were conducted to test the interactions of PM_2.5_ and green space on IS mortality.

**Results:**

A total of 10 799 IS deaths were included in our study. Exposure to PM_2.5_ was associated with an increased risk of IS mortality, with odds ratios (ORs) of 1.0263 (95% confidence interval (CI) = 1.0017, 1.0516) for each interquartile range (IQR) increase in PM_2.5_ on lag0 and 1.0317 (95% CI = 1.0016, 1.0627) on lag01. The links between PM_2.5_ and IS mortality were not significantly different across genders, ages, or PM_2.5_ zones. Furthermore, our results showed that the effects of PM_2.5_ on IS mortality were higher in low levels of green space. Specifically, for each IQR increase in PM_2.5_, the ORs (95% CIs) of IS death in the low level and the high level of NDVI were 1.0287 (95% CI = 1.0019, 1.0563) and 0.9934 (95% CI = 0.9296, 1.0615), respectively. In addition, PM_2.5_ and NDVI exhibited significant interactive effects on IS mortality, with relative excess odds due to interaction (REOI) of greater than 0.

**Conclusions:**

Our findings showed that PM_2.5_ was significantly associated with increasing odds of IS mortality. Furthermore, there were synergetic impacts between PM_2.5_ and lack of greenness on IS mortality. Our results suggest that expanding green spaces, such as increasing park coverage and street greening, along with regulating industrial emissions to reduce PM_2.5_ levels, can help prevent premature deaths from IS.

Stroke is a major cause of mortality, particularly in low- and middle-income countries [1,2]. Ischemic stroke (IS) is a primary subtype of stroke and is mainly characterised by vascular stenosis and occlusion, typically resulting from thrombotic or embolic events [3]. According to the 2019 Global Burden of Disease (GBD 2019) study, in 204 countries and regions worldwide, China ranked first in terms of the number of IS deaths [4]. In 2019, the crude mortality rate of IS in China was 72.4 per 100 000, representing a 125.6% increase compared to that in 1990 [4]. Therefore, implementing effective prevention strategies is crucial for alleviating the burden of IS in China. Although conventional risk factors, such as tobacco use, hypertension, hyperlipidaemia, diet, and physical activity, contribute substantially to IS mortality [5], growing evidence suggests that environmental risk factors could also increase the risk of IS mortality [6,7].

The GBD 2019 estimated that ambient particulate matter was a serious risk factor for stroke deaths, and the age-standardised death rate for IS attributed to air pollutants among Chinese residents was 17.1 per 100 000 in 2019 [4]. Fine particulate matter (PM_2.5_) is a primary component of ambient particulate matter and poses considerable health hazards to humans [8]. Several studies have shown that inhalation of PM_2.5_ can lead to endothelial injury and dysfunction [9], triggering systemic inflammation and thrombosis [10], which in turn causes changes in cerebrovascular haemodynamics, enhances blood coagulability, and increases plasma viscosity [11], ultimately raising the risk of IS mortality. To date, some epidemiological research has reported associations between short-term exposure to PM_2.5_ and IS mortality [7,12]. However, previous studies have reported mixed and inconclusive results, which may stem from variations in study design, study location, pollution levels, and population characteristics [6]. Additionally, in the past decade, with the introduction of policies aimed at preventing and controlling air pollution, the air quality in China has improved [13]. However, with the accelerated pace of urbanisation, China's pollution problem remains fundamentally challenging to change in the short term. For example, over a substantial portion of the period from 2015 to 2019, certain regions in China, including Zibo City, experienced PM_2.5_ concentrations exceeding the World Health Organization's (WHO) interim target of 35 µg per cubic metre (μg/m^3^) [14]. Given the severe air pollution [15] and the high IS mortality burden [4] in China, further research is necessary to enhance our understanding of the impact of PM_2.5_ on IS mortality.

Apart from PM_2.5_, it is plausible that there is a link between green space and IS mortality [16]. Green space, which can enhance the ecological environment, health, and overall quality of life for individuals, is commonly described as undeveloped, open land with natural vegetation [17]. Many studies have reported that green space has various health benefits for humans, such as reducing the mortality risk of cardiovascular diseases [18,19]. Furthermore, an increasing number of studies have suggested that the impact of environmental exposures on health outcomes is not separate but rather the synergistic impact of mixed exposures [20,21]. The combined impact of PM_2.5_ and green space may have more profound implications for humans than their independent impacts [22]. For example, two studies conducted in China reported that green space could reduce the risk of air pollution-related mortality [22,23]. Current research has reported a link between PM_2.5_ and IS mortality [6,7], but whether PM_2.5_ and green space can interact to precipitate IS deaths is still unknown. In China, rapid urbanisation has resulted in a shift in which more people live in urban areas, which have worse air quality and fewer green space areas [24,25]. Therefore, considering the limited number of related studies and the complex situation of simultaneous exposure to PM_2.5_ and green space, examining the combined impact of PM_2.5_ and green space on IS mortality is intriguing and crucial.

This research aimed to estimate relationships between PM_2.5_ and IS mortality and evaluate the role of green space in the association using time-series data on IS mortality in Zibo City, China, from 2015 to 2019.

## METHODS

### Study setting

Zibo City is located between latitude 35°55'20” and 37°17'14” north and longitude 117°32'15” and 118°31'00” east and is situated in the central part of Shandong Province and the eastern region of China (Figure S1 in the **Online Supplementary Document**). Zibo City is in a warm temperate zone, with an annual average temperature ranging from 12.5°C to 14.2°C and an average yearly precipitation of 650 mm. This city is an important industrial centre with a strong presence in industries such as petrochemicals and textiles. Furthermore, the city's green space area has been expanding, reaching over 13 000 hectares by the end of 2018. The urban green coverage rate is 44.78%, and the per capita park green space area is 20.39 m^2^. According to the 2020 Census, Zibo's population is 4.7 million, with 16.50% of residents aged 65 or older (http://www.zibo.gov.cn/).

### Data collection

Daily mortality counts of IS in Zibo City from 1 January 2015 to 31 December 2019, were obtained from the Shandong Province chronic disease and cause of death monitoring comprehensive management information system. For all deaths, we collected details on the date of death, causes of death, gender, age, and residential address. According to the Tenth Revision of the International Statistical Classification of Diseases and Related Health Problems, I63 was chosen as the diagnostic code for IS. The identifiable personal information has been removed to ensure privacy protection. The data were cleaned and assessed for quality, including checking for any misclassification of gender, age, and cause of death codes, as well as deaths that did not belong to the study area or study period. Finally, one case with gender discordance and 125 cases with residential addresses outside of Zibo City were excluded.

The daily PM_2.5_, sulphur dioxide (SO_2_), and nitrogen dioxide (NO_2_) concentrations covering Zibo City during our study period were collected from the China High Air Pollutants (CHAP, https://weijing-rs.github.io/product.html) data set. Artificial intelligence models were used to create the CHAP data set based on the spatiotemporal heterogeneity of air pollutants. This data set provided comprehensive spatiotemporal coverage across Zibo City during the study period, with a spatial resolution of 1 × 1 km for PM_2.5_ and 10 × 10 km for SO_2_ and NO_2_. The cross-validated coefficient of determination (R^2^) for PM_2.5_, SO_2_, and NO_2_ was 0.92, 0.84, and 0.84, respectively, with corresponding root-mean-square errors (RMSE) of 10.76 μg/m^3^, 10.07 μg/m^3^, and 7.99 μg/m^3^ [26–28].

The normalised difference vegetation index (NDVI) is designed to evaluate the global distribution of vegetation types and is commonly used in studies to assess the effect of green spaces on health outcomes due to its widespread applicability, relatively high spatial resolution, and consistency in objectively measuring green spaces [29]. Monthly NDVI data covering Zibo City during our study period were obtained from the Moderate Resolution Imaging Spectroradiometer (MODIS) data repository (https://modis.gsfc.nasa.gov/). The MODIS data repository comprises monthly NDVI data with a 1 × 1 km spatial resolution. The range of NDVI values was between −1 and 1, where higher values indicate a greater extent of green vegetation cover. The MODIS products have been validated through extensive ground truthing and accuracy assessments across various locations and periods and have been widely used in previous scientific studies [21,30].

Meteorological data, including hourly temperature (TEM) and hourly dewpoint temperature (DT), were sourced from the European Centre for Medium-Range Weather Forecasts (ECMWF, https://cds.climate.copernicus.eu/) [31]. The ERA5-Land data set was generated by repeating the land component of the ECMWF ERA5 climate reanalysis. It provides hourly meteorological data with a spatial resolution of 0.25° × 0.25° for the atmosphere, ocean, and land surface [31]. The hourly relative humidity (RH) was calculated as follows [32]:



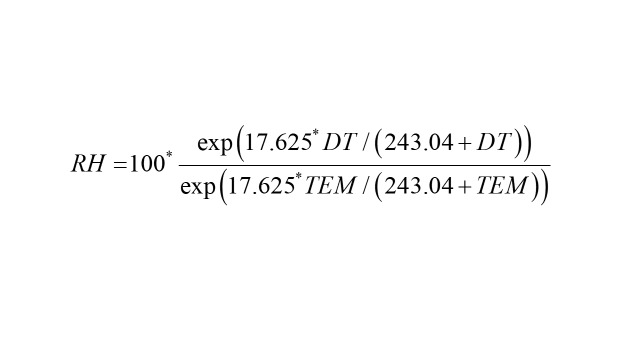



Where *RH* indicates the relative humidity, *TEM* indicates the mean temperature, and *DT* indicates the dew point temperature. The daily TEM and RH within each grid in Zibo City were the arithmetic means of the hourly data.

During our study period, daily PM_2.5_, SO_2_, NO_2_, temperature, relative humidity, and monthly NDVI in Zibo City were determined by computing the mean values of all pixels within the region [16,33].

### Statistical analysis

First, single-day lag models (lagN, N = 0, 1, 2... 5) and cumulative lag models (lag0N, N = 1, 2...5) were used to examine the impacts of short-term exposure to PM_2.5_ on IS mortality. Odds ratios (ORs) with 95% confidence intervals (CIs) for IS mortality were adopted to evaluate the effects of each interquartile range (IQR) increase in the PM_2.5_ concentration. The model was described as follows:

*log* [*E* (*Y_t_*)] = α + *PM_2.5_* (*lagN or lag0N*) + *ns*(*TEM07,3*) + *ns*(*RH07,3*) + *ns*(*DOY,3*) + *YEAR* + *HOLIDAY* + *DOW*

Where *Y_t_* represents daily IS deaths, following a quasi-Poisson distribution. *PM_2.5_* (*lagN or lag0N*) indicates the concentration of PM_2.5_ on different lag periods. For instance, lag1 represents the concentration of PM_2.5_ on the day before death, and lag01 represents the average concentration of PM_2.5_ on the day of death and the day before death. *ns* () represents the natural cubic spline function. *TEM07* and *RH07* represented the seven days cumulative lag average temperature and RH, with degrees of freedom (dfs) set to 3 [33]. *DOY* is the day of the year to control for seasonality, and the value ranges from 1 to 365 or 366, with dfs set to 3 [34]. *YEAR* is the year of death to control for long-term trends. *HOLIDAY* considers holidays to control for holiday effects. *DOW* represents the day of the week to control for day-of-week effects. The dfs were set based on prior literature knowledge and the results of generalised cross-validation [35,36].

Subgroup analyses by gender (male, female) and age (<75 years, ≥75 years) and PM_2.5_ levels (high PM_2.5_ zone/low PM_2.5_ zone, based on the median) were implemented to identify vulnerable populations. We estimated the effects of PM_2.5_ on IS mortality in different subgroups. A two-sample Z-test was employed to examine the differences in the estimates of subgroup-specific effects for each categorical variable [37].



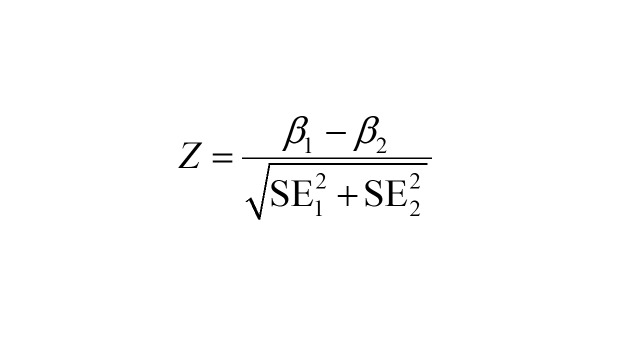



Where β_1_ and β_2_ represent the estimated effects of PM_2.5_ in two compared subgroups, while SE_1_ and SE_2_ represent the respective standard errors for β_1_ and β_2_.

Second, stratified analyses by green space levels were conducted to test the modifying effects of green space. The NDVI was classified into two categories with centroids of 0.31 and 0.57, and into three categories with centroids of 0.28, 0.46, and 0.65, using the K-means clustering method. Additional stratification was performed based on different percentiles (P25, P50, and P75) of NDVI. If the effects of PM_2.5_ on lag0 on IS mortality differ in different levels of NDVI, it indicates green space modified the association between PM_2.5_ with IS mortality.

In the third step, we used a joint effect model to estimate the combined effects of co-exposure to PM_2.5_ on lag0 and NDVI on IS mortality. We categorised PM_2.5_ concentrations as binary variables based on the least stringent Interim Target specified by the WHO (35 μg/m^3^), the mean concentration of PM_2.5_, and different percentiles (P50 and P75) of concentration of PM_2.5_. The NDVI was categorised into binary variables based on different percentiles (P25, P50, and P75) of NDVI, respectively. We created a new categorical variable, PN, to serve as the amalgamation of two binary variables, which comprised four levels:

1)low-level of PM_2.5_ and high-level of NDVI (level 1, reference group);

2)high-level of PM_2.5_ and high-level of NDVI (level 2);

3)low-level of PM_2.5_ and low-level of NDVI (level 3);

4)high-level of PM_2.5_ and low-level of NDVI (level 4).

The model was described as follows:

log [*E* (*Y_t_*)] = α + *PN* + *ns* (*TEM01,3*) + *ns* (*RH01,3*) + *ns* (*DOY,3*) + *YEAR* + *HOLIDAY* + *DOW*

Where *Y_t_* represents daily IS deaths, following a quasi-Poisson distribution. *PN* is the newly formed variable PN. Other control variables and parameter settings were the same as above. Addictive interactions were examined using the relative excess odds due to interaction (REOI). The REOI was calculated as follows [38,39]:

REOI = (OR_11_ − 1) − (OR_10_ − 1) − (OR_01_ − 1) = OR_11_ − OR_10_ − OR_01_ + 1)

Where OR_10_, OR_01_, and OR_11_ represent the ORs at levels 2, 3, and 4 compared with level 1 (OR_00_ = 1), respectively. The corresponding 95% CIs for REOI were calculated using the delta method [40]. When the 95% CIs of REOI include 0, the interactive effects are not statistically significant. When the 95% CIs of REOI exclude 0 and are greater than 0, this indicates a synergistic interaction. When the 95% CIs of REOI exclude include 0 and are less than 0, there is a negative interaction.

In addition, to quantify excess mortality attributed to co-exposure to PM_2.5_ and NDVI, we calculated excess fraction (EF) and number of excess deaths (ED) based on exposure level 4 [20,41].



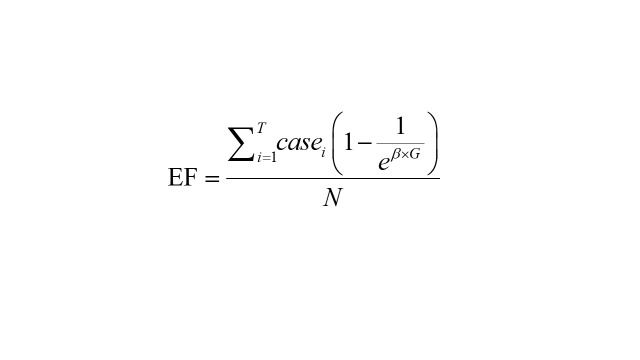





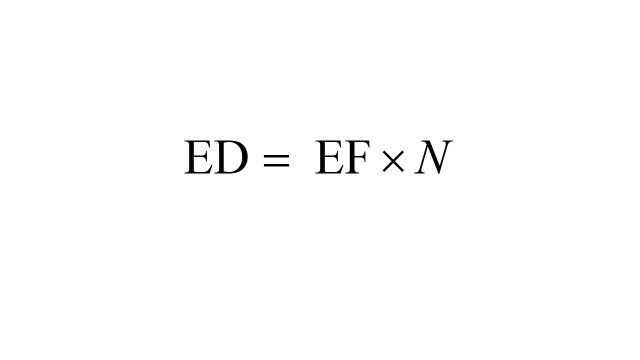



Where β indicates the effect values of exposure level 4; *G* denotes whether the exposure on the day of death was exposure level 4 (1 = yes, 0 = no); *N* and *case_i_* indicate the overall number of IS deaths and daily IS deaths, respectively; *T* indicates the overall number of days in the study period.

### Sensitivity analysis

First, to explore the nonlinear impacts of PM_2.5_ on IS mortality, we fitted PM_2.5_ as smoothing terms by employing restricted cubic splines with three knots at the 10th, 50th, and 90th percentiles of the values [42]. Second, we introduce other air pollutants (*i.e*. SO_2_ and NO_2_) into models to evaluate the robustness of our findings. Finally, we used PM_2.5_ on lag01 as the exposure indicator to test the modified effects of green space and evaluate the interactive effects of PM_2.5_ and NDVI.

All analyses were performed using *R*, version 4.3.1 (R Core Team, Vienna, Austria), and statistical significance was defined as a two-sided *P*-value less than 0.05.

## RESULTS

A total of 10 799 IS deaths in Zibo City were reported from 2015 to 2019, with an average of 5.91 deaths per day (**Table 1**). Among the subjects, 65.24% of deaths were older adults (aged ≥75), and 52.70% were males. The medians of PM_2.5_ and NDVI were 49.60 μg/m^3^ and 0.43, respectively, with IQR of 36.3 μg/m^3^ and 0.31.

**Table 1:** Daily number of ischemic stroke (IS) deaths and environmental factors in Zibo City, China (2015–2019)

Factor	Mean±SD	Percentiles				
		**Min**	**P_25_**	**Med**	**P_75_**	**Max**
Death cases	5.91 ± 2.79	0.00	4.00	6.00	8.00	17.00
Age ≥75	3.86 ± 2.20	0.00	2.00	4.00	5.00	13.00
Age <75	2.06 ± 1.50	0.00	1.00	2.00	3.00	9.00
Male	3.12 ± 1.91	0.00	2.00	3.00	4.00	12.00
Female	2.80 ± 1.80	0.00	1.00	3.00	4.00	11.00
PM_2.5_ (μg/m^3^)	58.25 ± 34.72	6.60	34.70	49.60	71.00	295.8
SO_2_ (μg/m^3^)	42.60 ± 28.44	5.19	21.68	34.65	55.91	190.22
NO_2_ (μg/m^3^)	41.68 ± 15.04	11.85	30.43	40.32	50.53	114.89
NDVI	0.43 ± 0.15	0.22	0.30	0.43	0.61	0.70
TEM (°C)	13.51 ± 10.44	−12.70	3.84	15.14	22.86	30.94
RH (%)	58.13 ± 17.24	16.73	44.76	57.79	71.44	98.58

Max – maximum, Med – median, Min – minimum, NDVI – normalised difference vegetation index, NO_2_ – nitrogen dioxide, PM_2.5_ – fine particulate matter, P_25_ – 25th percentile, P_75_ – 75th percentile, RH – relative humidity, SD – standard deviation, SO_2_ – sulphur dioxide, TEM – temperature, μg/m^3^ – micrograms per cubic metre

**Figure 1:**
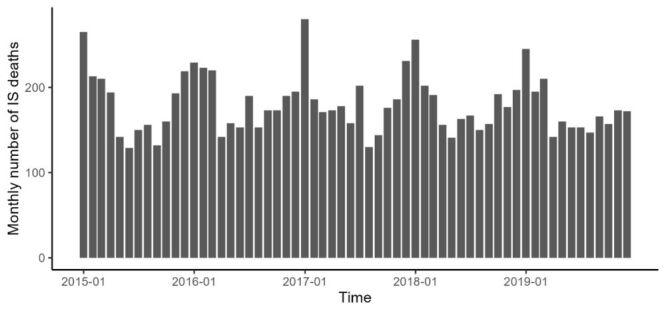
The time series distribution of monthly ischemic stroke (IS) deaths in Zibo, Shandong Province, 2015–2019.

The temporal distribution of IS deaths exhibited distinct periodicity and seasonality, with an annual peak occurring mainly during the cold season (October to March of each year) (**Figure 1**). PM_2.5_ concentrations were higher in the cold and lower in the warm seasons (April to September of each year) (Figure S2 in the **Online Supplementary Document**). The opposite was true for NDVI.

**Figure 2:**
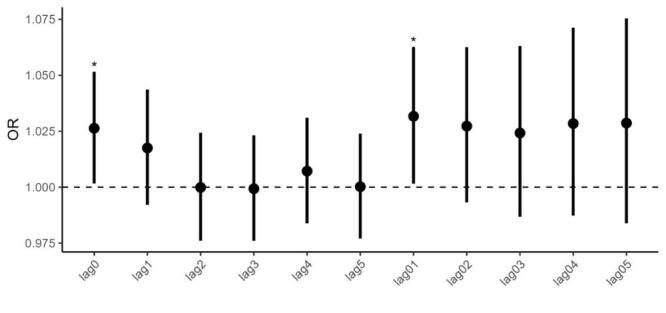
Associations between short-term exposure to fine particulate matter (PM_2.5_) and ischemic stroke (IS) mortality. *Indicates the corresponding *P*-value less than 0.05.

PM_2.5_ on lag0 and lag01 were significantly associated with IS mortality (**Figure 2**). For each IQR increase in PM_2.5_ on lag0 and lag01, the ORs (95% CIs) of IS mortality were 1.0263 (95% CI = 1.0017, 1.0516) and 1.0317 (95% CI = 1.0016, 1.0627), respectively. The links between PM_2.5_ and IS mortality were not significantly different across genders, ages, or PM_2.5_ zones (Figure S3–4 in the **Online Supplementary Document**).

**Figure 3:**
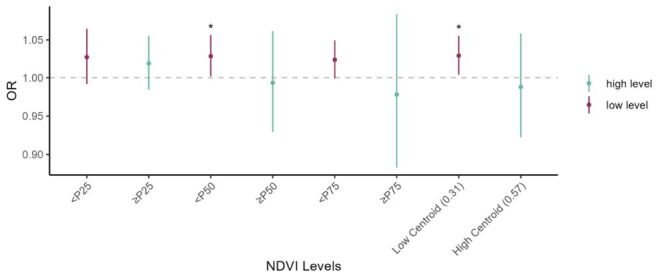
Associations between fine particulate matter (PM_2.5_) concentration on lag0 and ischemic stroke (IS) mortality across different green space levels. The low centroid and high centroid represent the two cluster centres of normalised difference vegetation index (NDVI) values, with values of 0.31 and 0.57, respectively. The values in parentheses indicate the centroid of each class.

The effects of PM_2.5_ on IS mortality were higher in low levels of green space (**Figure 3**; Figure S5 in **the** **Online Supplementary Document**). For example, under the stratified analysis based on P50 of NDVI, for each IQR increase in PM_2.5_, the ORs (95% CIs) of IS mortality in the low level and the high level of NDVI were 1.0287 (95% CI = 1.0019, 1.0563) and 0.9934 (95% CI = 0.9296, 1.0615), respectively.

PM_2.5_ on lag0 and NDVI exhibited significant interactive effects on IS mortality (**Table 2**; Table S1 in the **Online Supplementary Document**). Under classified standards of the mean of PM_2.5_ and P50 of NDVI, P75 of PM_2.5_ and P50 of NDVI, PM_2.5_ and NDVI exhibited significant interactive effects on IS mortality, with REOIs of 0.13 (95% CI = 0.03, 0.22) and 0.15 (95% CI = 0.01, 0.28), respectively. The corresponding attributable fractions of excess mortality and the excess deaths for combined exposure level 4 were 2.84% (306.69 excess deaths) and 2.32% (250.54 excess deaths), respectively (**Table 3**).

**Table 2:** Additive interactive effects of fine particulate matter (PM_2.5_) on lag0 and normalised difference vegetation index (NDVI) on IS mortality

Classified standards of PM_2.5_ and NDVI	PM_2.5_/NDVI	No. of death cases (%)	ORs (95%CIs)
Least stringent interim target of PM_2.5_ (35 μg/m^3^)			
P50 of NDVI (0.43)	Low-high	1902 (17.61)	Ref.
	High-high	2652 (24.56)	0.99 (0.92, 1.06)
	Low-low	598 (5.54)	1.01 (0.90, 1.13)
	high-low	5647 (52.29)	1.03 (0.95, 1.13)
REOI			0.05 (−0.07, 0.16)
Median of PM_2.5_ (49.60 μg/m^3^)			
P50 of NDVI (0.43)	Low-high	3288 (30.45)	Ref.
	High-high	1266 (11.72)	0.97 (0.90, 1.05)
	Low-low	1698 (15.72)	1.00 (0.92, 1.09)
	High-low	4547 (42.11)	1.06 (0.97, 1.15)
REOI			0.09 (−0.01, 0.18)
Mean of PM_2.5_ (58.25 μg/m^3^)			
P50 of NDVI (0.43)	Low-high	3779 (34.99)	Ref.
	High-high	775 (7.18)	0.96 (0.88, 1.05)
	Low-low	2536 (23.48)	1.00 (0.93, 1.09)
	High-low	3709 (34.35)	1.09 (1.00, 1.18)
REOI			0.13* (0.03, 0.22)
P75 of PM_2.5_ (71.00 μg/m^3^)			
P50 of NDVI (0.43)	Low-high	4219 (39.07)	Ref.
	High-high	335 (3.10)	0.93 (0.83, 1.05)
	Low-low	3489 (32.31)	1.02 (0.95, 1.10)
	High-low	2756 (25.52)	1.10 (1.01, 1.19)
REOI			0.15* (0.01, 0.28)

CI – confidence intervals, NDVI – normalised difference vegetation index, PM_2.5_ – fine particulate matter, OR – odds ratio, REOI – relative excess odds due to interaction, μg/m^3^ – micrograms per cubic metre

*Significant interactive effects of PM_2.5_ and NDVI.

**Table 3:** Excess mortality attributed to co-exposure to exposure levels 4

Exposure levels of PM_2.5_ and NDVI	Excess fraction (%)	No. of excess deaths
PM_2.5_≥35.00 μg/m^3^ and NDVI<0.30	4.30	464.35
PM_2.5_≥49.60 μg/m^3^ and NDVI<0.30	4.15	448.16
PM_2.5_≥58.25 μg/m^3^ and NDVI<0.30	2.99	322.89
PM_2.5_≥71.00 μg/m^3^ and NDVI<0.30	2.87	309.93
PM_2.5_≥35.00 μg/m^3^ and NDVI<0.43	1.50%	164.14
PM_2.5_≥49.60 μg/m^3^ and NDVI<0.43	2.39	258.10
PM_2.5_≥58.25 μg/m^3^ and NDVI<0.43	2.84	306.69
PM_2.5_≥71.00 μg/m^3^ and NDVI<0.43	2.32	250.54
PM_2.5_≥35.00 μg/m^3^ and NDVI<0.61	0.00	0.00
PM_2.5_≥49.60 μg/m^3^ and NDVI<0.61	0.98	105.83
PM_2.5_≥58.25 μg/m^3^ and NDVI<0.61	1.50	161.99
PM_2.5_≥71.00 μg/m^3^ and NDVI<0.61	1.05	113.39

NDVI – normalised difference vegetation index, PM_2.5_ – fine particulate matter, μg/m^3^ – micrograms per cubic metre

In sensitivity analysis, linear correlations were observed between PM_2.5_ on lag0 and lag01 with IS mortality (Figure S6 in the **Online Supplementary Document**). Sensitivity analyses by adjusting for SO_2_ and NO_2_ in the models (Figure S7 in the **Online Supplementary Document**) and using PM_2.5_ on lag01 as the exposure indicator (Figure S8 and Table S2–3 in the **Online Supplementary Document**) gave similar results.

## DISCUSSION

This study conducted a time-series study of 10 799 IS deaths in Zibo City from 2015 to 2019. We comprehensively investigated the associations between short-term exposure to PM_2.5_ and IS mortality. In addition, we tested the modified impacts of green space on the associations and the additive interactions of PM_2.5_ and NDVI on IS mortality. Our data found that PM_2.5_ on lag0 and lag01 could significantly raise the risk of IS mortality. Furthermore, the effects of PM_2.5_ on IS mortality were higher in low levels of green space. In addition, we observed significant synergistic effects of PM_2.5_ and NDVI on IS mortality, and IS mortality was most attributed to simultaneous exposure to the high level of PM_2.5_ and the low level of NDVI.

The results presented here demonstrated that PM_2.5_ was significantly linked with an increased likelihood of IS mortality. Our research suggested that mitigating the ambient PM_2.5_ concentration is critical for the reduction of IS mortality. This finding aligns with the outcomes observed in previous research [6,7]. For instance, Ban et al. found that, with a 10 μg/m^3^ increase in PM_2.5_, the IS mortality risk increased by 1.09% (95% CI = 0.05%, 2.14%) [43]. A prospective cohort study in 161 districts/counties across China found that the hazard ratio of IS mortality per 10 μg/m^3^ increase in PM_2.5_ concentration was 1.11 (95% CI = 1.04, 1.19) [44]. However, some studies conducted in China [45] and the US [46,47] have reported no statistically significant associations between short-term exposure to PM_2.5_ and IS mortality. These discrepancies may be due to differences in study design, location, pollution levels, and population characteristics. Moreover, it is biologically plausible that PM_2.5_ could acutely trigger IS deaths [6]. Exposure to PM_2.5_ has been demonstrated to increase the expression of markers of plaque vulnerability, specifically matrix metalloproteinases, and to induce systemic inflammation and thrombogenicity [10]. PM_2.5_ can also initiate an autonomic reflex through pulmonary receptors, baroreceptors, and chemical receptors, leading to cardioembolic ischemic stroke attributed to cardiac ischemia or cardiac arrhythmia [48]. We found that older adults were more vulnerable to PM_2.5_ on lag0, although the difference was not statistically significant. Similar results have been reported in previous studies [6], which may be related to the more fragile cardiovascular structure and function in older adults [49]. Additionally, older adults often have other cardiovascular risk factors that may increase the risk of air pollution-related stroke [6]. We found similar effect sizes for men and women in terms of PM_2.5_-related ischemic stroke mortality. Women have smaller airway diameters, which makes it easier for particulate matter to deposit in their lungs [50], while men tend to spend more time outdoors, leading to greater exposure to outdoor air pollution [51]. Therefore, the gender differences in the effects of PM_2.5_ still require further research. We also found that individuals living in regions with high levels of PM_2.5_ at lag0 had an increased risk of IS mortality, although the difference was not statistically significant. It is advisable to pay closer attention to populations living in high-pollution areas.

The effects of PM_2.5_ on IS mortality were higher in low levels of green space, and there were interactions between PM_2.5_ and NDVI on IS mortality. We suggest that mitigating PM_2.5_ and increasing greening may contribute to preventing premature death from IS. A growing body of literature has explored the combined effects of PM_2.5_ and NDVI on adverse health outcomes. For instance, Ji et al. found that greenness and air pollutants synergistically affected mortality. For every 0.1 reduction in NDVI and per 10 μg/m^3^ increase in PM_2.5_, the hazard ratio of the product term was 1.01 (95% CI = 1.00, 1.02) [23]. Sun et al. found that decreasing NDVI (a 250-m buffer) and high levels of air pollution had positive additive effects on preterm birth, with an REOI of 0.009 (95% CI = 0.003, 0.016) [52]. Yang et al. reported that PM_2.5_ played a substantial mediating role, accounting for 37.0% of the relationship between green space and the odds of incidence of type 2 diabetes [53]. A study conducted in 10 provinces of China showed that green space could alleviate the harmful impact of PM_2.5_ on metabolic syndrome (OR = 0.988; 95% CI = 0.984, 0.993) [54]. Guo et al. found that PM_2.5_ could mediate the estimated relationship between residential greenness and frailty [55]. A UK-wide cohort study found that greening can modulate the association between PM_2.5_ and physiological stress [56]. A study from Denmark found that adjusting for NDVI increased the mortality hazard ratios for PM_2.5_ in 1990 [57]. However, despite growing interest in analysing the combined effects of PM_2.5_ and NDVI, few studies have examined their interaction on IS mortality. To our knowledge, this study is among the few studies in the literature systematically quantifying the interaction of PM_2.5_ and NDVI on IS mortality. Our data identified a significant interaction between PM_2.5_ and NDVI, with the IS mortality most attributed to simultaneous exposure to the high level of PM_2.5_ and the low level of NDVI. The combined impact between PM_2.5_ and green space is multi-mechanistic [58]. Green space and vegetation can aerodynamically intercept airborne particles, with particles either retained on leaf surfaces or absorbed into trees [59,60]. Otherwise, it is biologically plausible that green space could alter the inherent properties of PM_2.5_, such as its size. These alterations may expedite the deposition of PM_2.5_ and alter its composition, which could reduce its concentration and toxicity [58,61,62]. Therefore, urban planners should prioritise implementing green initiatives to reduce the public health risks associated with air pollution. For urbanised regions facing air pollution issues and greening challenges, promoting urban greening, such as street greening and expanding park coverage, and strengthening industrial emissions regulation to lower PM_2.5_ concentrations, which would help reduce stroke mortality.

Our study has several limitations. First, due to resource constraints, we used the daily concentrations of PM_2.5_ and NDVI for the entire city to represent individual exposure levels, which may have led to exposure misclassification bias. Second, as an ecological study, causal relationships between PM_2.5_ and IS mortality cannot be established. In addition, our study did not consider factors such as socioeconomic status, health care access, individual comorbidities, and lifestyle behaviours. To enhance the reliability of the findings, we recommend that future data collection efforts specifically focus on gathering relevant individual-level data. Third, the main effects of PM_2.5_ on IS mortality are small. However, they are statistically significant, suggesting they are unlikely to be due to chance. In epidemiological research, small effects can still be meaningful, particularly in large populations, where even minor changes in risk can translate into significant public health impacts. Finally, this study is designed within a single region in China, thus limiting the generalisability of the research findings.

## CONCLUSIONS

In summary, PM_2.5_ was significantly linked to increased odds of IS mortality, especially in lower levels of green space. Furthermore, there were synergetic impacts between PM_2.5_ and lack of greenness on IS mortality. Our findings suggest that expanding green spaces, such as increasing park coverage and street greening, along with regulating industrial emissions to reduce PM_2.5_ levels, can help prevent premature deaths from IS.

## Additional material


Online Supplementary Document
