# Supplementary material for: Associations between short-term exposure to fine particulate matter with ischemic stroke mortality and the role of green space: a time-series study in Zibo, China
Source: J Glob Health. 2025 Mar 21;15:04068. doi: 10.7189/jogh.15.04068 (PMC11927038; doi:10.7189/jogh.15.04068)
Supplement: Online Supplementary Document [file jogh-15-04068-s001.pdf]

Supplement to: Song S, Cheng C, Liu Y, Duan Y, Zuo H, Xi R, Ni Z, Liang K, Li S, Cui F, Li X. Associations between short-term exposure to fine particulate matter with ischemic stroke mortality and the role of green space: a time-series study in Zibo, China. J Glob Health. 2025;15:04068.

Figure and Table of Contents

Figure S1. The geographical location of Zibo City, Shandong Province..1

Figure S2. The time series distribution of air pollutants and NDVI in Zibo, Shandong Province, 2015-2019..1

Figure S3. Associations between short-term exposure to $PM_{2.5}$ and IS mortality across different ages and genders 2

Figure S4. Associations between short-term exposure to $PM_{2.5}$ and IS mortality across different $PM_{2.5}$ zones 2

Figure S5 Associations between $PM_{2.5}$ concentration on lag0 and IS mortality across 3 different green space levels 3

Table S1 Additive interactive effects of $PM_{2.5}$ on lag0 and NDVI on IS Mortality......................3-4

Figure S6. Exposure-response curves of associations between $PM_{2.5}$ concentration and IS mortality 4

Figure S7. Associations between short-term exposure to $PM_{2.5}$ and IS mortality with NO_2 and SO_2 were introduced in models..5

Figure S8. Associations between $PM_{2.5}$ concentration on lag01 and IS mortality across different green space levels..5

Table S2 Additive interactive effects of $PM_{2.5}$ on lag01 and NDVI on IS Mortality....................6-7

Table S3 Excess mortality attributed to coexposure to exposure levels 4 by using $PM_{2.5}$ on lag01 as the exposure indicator 8

Figure S9. Normal Q-Q plot for main model 8

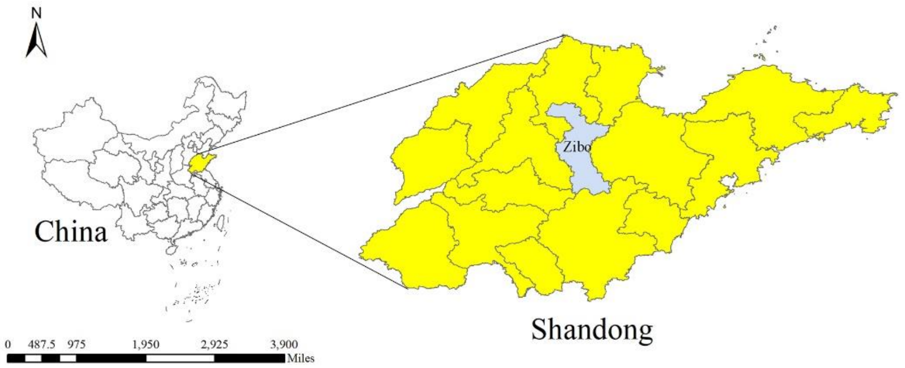

Figure S1. The geographical location of Zibo City, Shandong Province (the map was created with ArcMap software, 10.8).

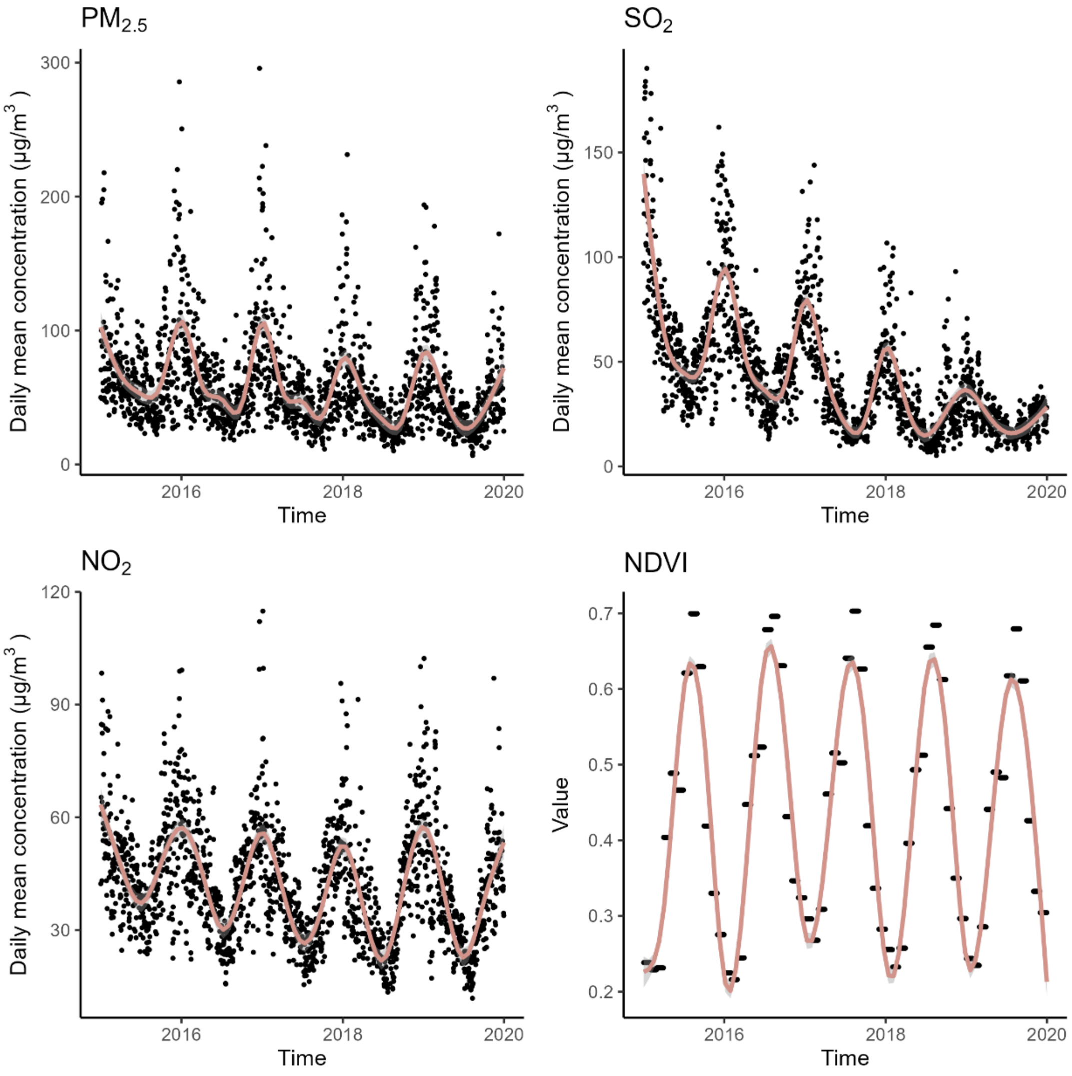

Figure S2. The time series distribution of air pollutants and NDVI in Zibo, Shandong Province, 2015-2019.

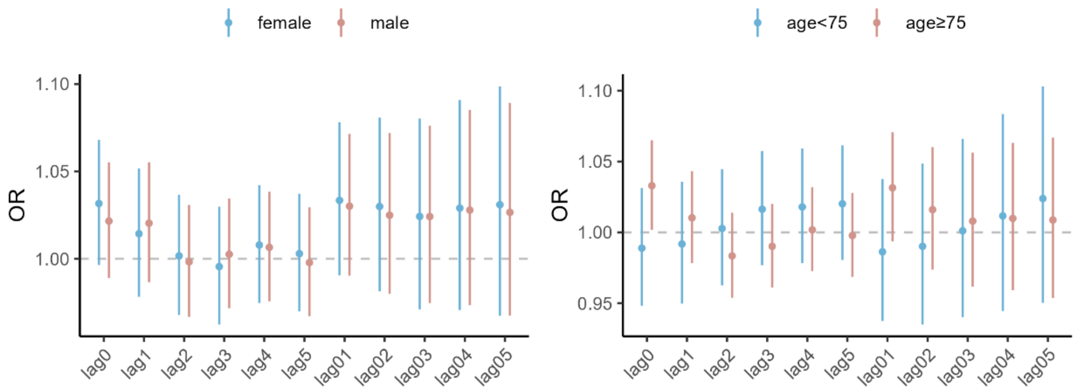

Figure S3. Associations between short-term exposure to $PM_{2.5}$ and IS mortality across different genders and ages.

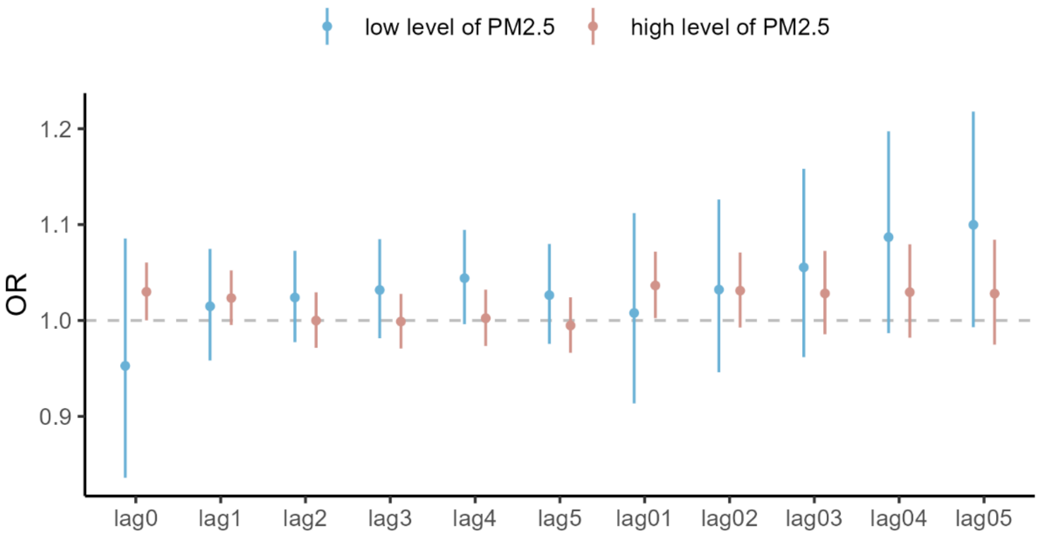

Figure S4. Associations between short-term exposure to $PM_{2.5}$ and IS mortality across different $PM_{2.5}$ zones.

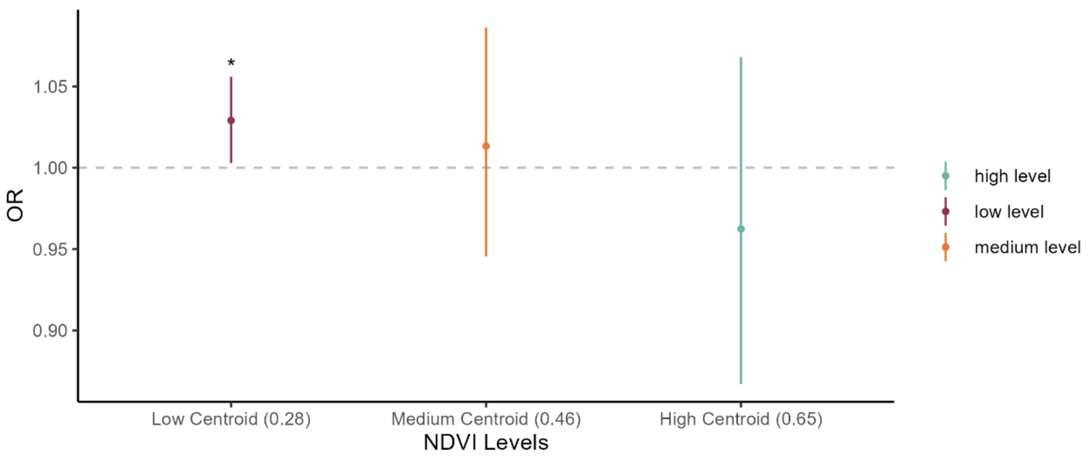

Figure S5. Associations between $PM_{2.5}$ concentration on lag0 and IS mortality across 3 different green space levels.

Note: * indicates the corresponding P-value < 0.05; The low centroid, medium centroid and high centroid represent the two cluster centers of NDVI values, with values of 0.28, 0.46 and 0.65, respectively.

Table S1 Additive interactive effects of $PM_{2.5}$ on lag0 and NDVI on IS Mortality

Classified standards of $PM_{2.5}$ and NDVI	$PM_{2.5}$-NDVI	Number of death cases	OR(95%CI)
Least stringent interim target of $PM_{2.5}$ (35 ug/m^3)			
P25 of NDVI (0.30)	low-high	2285 (21.16%)	Ref.
	high-high	4939 (45.74%)	1.00 (0.94, 1.06)
	low-low	215 (2.00%)	1.07 (0.90, 1.27)
	high-low	3360 (31.11%)	1.16* (1.05, 1.28)
REOI			0.09 (-0.08, 0.26)
Median of $PM_{2.5}$ (49.60 ug/m^3)			
P25 of NDVI (0.30)	low-high	4216 (39.04%)	Ref.
	high-high	3008 (27.85%)	1.01 (0.96, 1.07)
	low-low	770 (7.13%)	1.13* (1.01, 1.26)
	high-low	2805 (25.97%)	1.19* (1.08, 1.30)
REOI			0.05 (-0.06, 0.15)
Mean of $PM_{2.5}$ (58.25 ug/m^3)			
P25 of NDVI (0.30)	low-high	5139 (47.59%)	Ref.
	high-high	2085 (19.31%)	1.02 (0.96, 1.08)
	low-low	1176 (10.89%)	1.12* (1.01, 1.23)
	high-low	2399 (22.22%)	1.21* (1.10, 1.33)
REOI			0.07 (-0.03, 0.18)
P75 of $PM_{2.5}$ (71.00 ug/m^3)			
P25 of NDVI (0.30)	low-high	5991 (55.48%)	Ref.
	high-high	1233 (11.42%)	1.03 (0.96, 1.11)
	low-low	1717 (15.90%)	1.15* (1.05, 1.26)
	high-low	1858 (17.21%)	1.20* (1.09, 1.32)
REOI			0.02 (-0.09, 0.13)
Least stringent interim target of $PM_{2.5}$ (35 ug/m^3)			
P75 of NDVI (0.61)	low-high	1181 (10.93%)	Ref.
	high-high	1023 (9.47%)	1.01 (0.92, 1.11)
	low-low	1319 (12.21%)	1.00 (0.91, 1.11)
	high-low	7276 (67.38%)	1.00 (0.91, 1.10)
REOI			-0.01 (-0.12, 0.10)
Median of $PM_{2.5}$ (49.60 ug/m^3)			
P75 of NDVI (0.61)	low-high	1782 (16.50%)	Ref.
	high-high	422 (3.91%)	1.01 (0.90, 1.13)
	low-low	3204 (29.67%)	0.99 (0.91, 1.08)
	high-low	5391 (49.92%)	1.02 (0.93, 1.12)
REOI			0.02 (-0.11, 0.14)
Mean of $PM_{2.5}$ (58.25 ug/m^3)			
P75 of NDVI (0.61)	low-high	1939 (17.96%)	Ref.
	high-high	265 (2.45%)	1.01 (0.88, 1.16)
	low-low	4376 (40.52%)	0.99 (0.91, 1.08)

Classified standards of $PM_{2.5}$ and NDVI	$PM_{2.5}$-NDVI	Number of death cases	OR(95%CI)
	high-low	4219 (39.07%)	1.04 (0.95, 1.14)
REOI			0.05 (-0.12, 0.18)
P75 of $PM_{2.5}$ (71.00 ug/m^3)			
P75 of NDVI (0.61)	low-high	2072 (19.19%)	Ref.
	high-high	132 (1.22%)	0.94 (0.78, 1.14)
	low-low	5636 (52.19%)	0.99 (0.91, 1.08)
	high-low	2959 (27.40%)	1.04 (0.94, 1.15)
REOI			0.11 (-0.08, 0.30)

Note: The results of classifying NDVI using the K-means method are the same as those obtained using the P50 method.

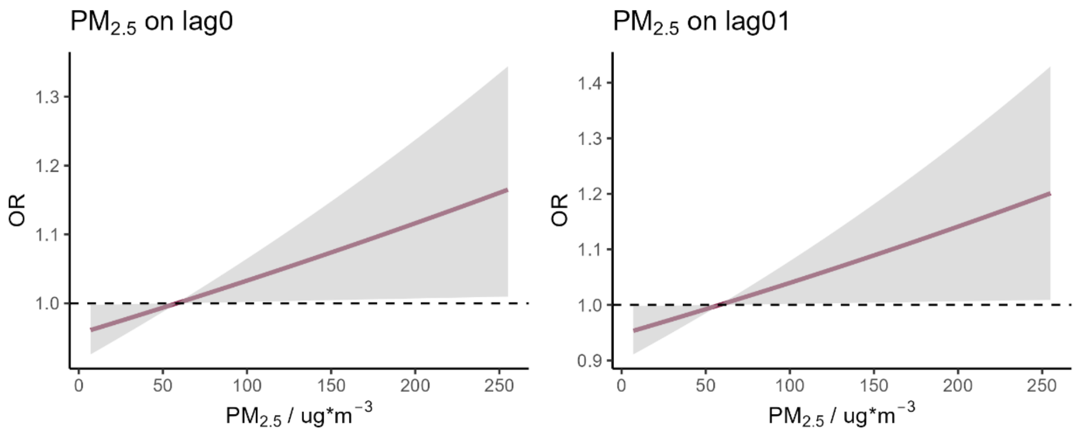

Figure S6. Exposure-response curves of associations between $PM_{2.5}$ concentration and IS mortality.

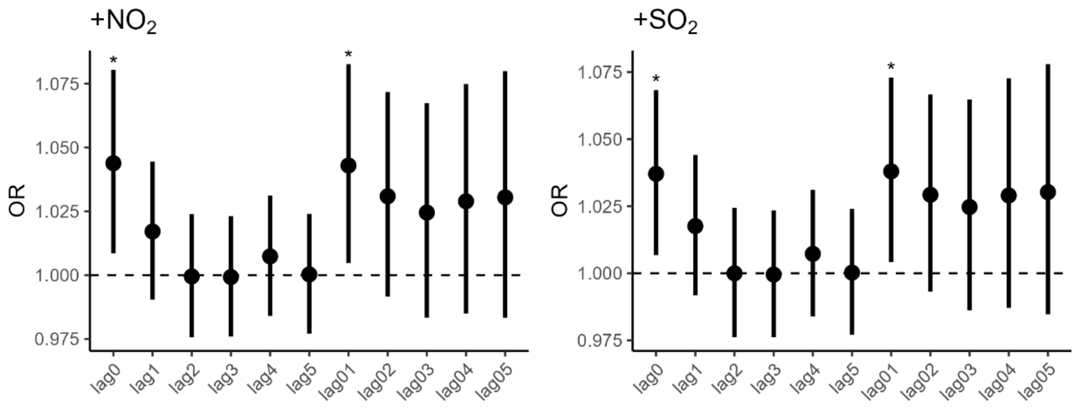

Figure S7. Associations between short-term exposure to $PM_{2.5}$ and IS mortality with NO_2 and SO_2 were introduced in models.

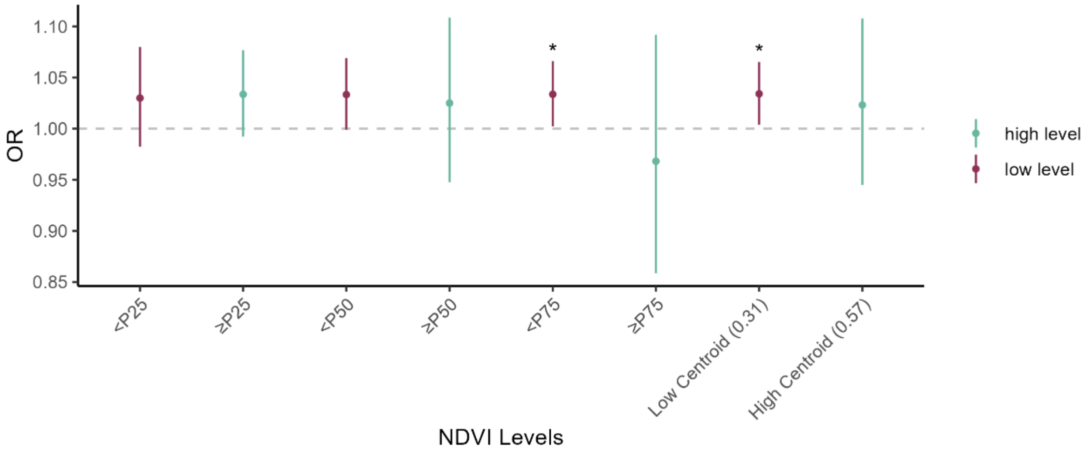

Figure S8. Associations between $PM_{2.5}$ concentration on lag01 and IS mortality across different green space levels.

Note: * indicates the corresponding P-value < 0.05; The low centroid and high centroid represent the two cluster centers of NDVI values, with values of 0.31 and 0.57, respectively. Using the K-means clustering method, we divided the NDVI values into two categories, with the low centroid corresponding to areas with lower NDVI values and the high centroid corresponding to areas with higher NDVI values. The values in parentheses indicate the centroid of each class.

Table S2 Additive interactive effects of $PM_{2.5}$ on lag01 and NDVI on IS Mortality

Classified standards of $PM_{2.5}$ and NDVI	$PM_{2.5}$-NDVI	Number of death cases	OR(95%CI)
Least stringent interim target of $PM_{2.5}$ (35 ug/m^3)			
P25 of NDVI (0.30)	low-high	1973 (18.27%)	Ref.
	high-high	5251 (48.62%)	1.02 (0.96, 1.08)
	low-low	141 (1.31%)	1.26* (1.01, 1.56)
	high-low	3434 (31.80%)	1.18* (1.06, 1.30)
REOI			-0.10 (-0.36, 0.15)
Median of $PM_{2.5}$ (49.60 ug/m^3)			
P25 of NDVI (0.30)	low-high	4207 (38.96%)	Ref.
	high-high	3017 (27.94%)	1.03 (0.97, 1.09)
	low-low	603 (5.58%)	1.14* (1.01, 1.29)
	high-low	2972 (27.52%)	1.19* (1.08, 1.31)
REOI			0.02 (-0.11, 0.15)
Mean of $PM_{2.5}$ (58.25 ug/m^3)			
P25 of NDVI (0.30)	low-high	5090 (47.13%)	Ref.

Classified standards of $PM_{2.5}$ and NDVI	$PM_{2.5}$-NDVI	Number of death cases	OR(95%CI)
	high-high	2134 (19.76%)	1.05 (0.98, 1.11)
	low-low	999 (9.25%)	1.14* (1.03, 1.27)
	high-low	2576 (23.85%)	1.22* (1.10, 1.34)
REOI			0.03 (-0.09, 0.14)
P75 of $PM_{2.5}$ (71.00 ug/m^3)			
P25 of NDVI (0.30)	low-high	6072 (56.23%)	Ref.
	high-high	1152 (10.67%)	1.02 (0.95, 1.10)
	low-low	1616 (14.96%)	1.11* (1.01, 1.22)
	high-low	1959 (18.14%)	1.24* (1.12, 1.36)
REOI			0.11 (-0.01, 0.22)
Least stringent interim target of $PM_{2.5}$ (35 ug/m^3)			
P50 of NDVI (0.43)	low-high	1695 (15.70%)	Ref.
	high-high	2859 (26.47%)	1.01 (0.94, 1.09)
	low-low	419 (3.88%)	1.07 (0.94, 1.21)
	high-low	5826 (53.95%)	1.04 (0.95, 1.15)
REOI			-0.04 (-0.17, 0.10)
Median of $PM_{2.5}$ (49.60 ug/m^3)			
P50 of NDVI (0.43)	low-high	3276 (30.33%)	Ref.
	high-high	1278 (11.83%)	0.99 (0.92, 1.06)
	low-low	1534 (14.21%)	1.00 (0.92, 1.09)
	high-low	4711 (43.62%)	1.07 (0.98, 1.16)
REOI			0.08 (-0.01, 0.18)
Mean of $PM_{2.5}$ (58.25 ug/m^3)			
P50 of NDVI (0.43)	low-high	3792 (35.11%)	Ref.
	high-high	762 (7.06%)	0.98 (0.89, 1.06)
	low-low	2297 (21.27%)	1.00 (0.93, 1.09)
	high-low	3948 (36.56%)	1.10* (1.00, 1.20)
REOI			0.12* (0.02, 0.22)
P75 of $PM_{2.5}$ (71.00 ug/m^3)			
P50 of NDVI (0.43)	low-high	4284 (39.67%)	Ref.
	high-high	270 (2.50%)	0.92 (0.80, 1.05)
	low-low	3404 (31.52%)	1.02 (0.95, 1.10)
	high-low	2841 (26.31%)	1.13* (1.03, 1.24)
REOI			0.19* (0.05, 0.33)
Least stringent interim target of $PM_{2.5}$ (35 ug/m^3)			
P75 of NDVI (0.61)	low-high	1116 (10.33%)	Ref.
	high-high	1088 (10.08%)	1.00 (0.91, 1.09)
	low-low	998 (9.24%)	0.99 (0.89, 1.10)

Classified standards of $PM_{2.5}$ and NDVI	$PM_{2.5}$-NDVI	Number of death cases	OR(95%CI)
	high-low	7597 (70.35%)	1.00 (0.91, 1.11)
REOI			0.01 (-0.10, 0.13)
Median of $PM_{2.5}$ (49.60 ug/m^3)			
P75 of NDVI (0.61)	low-high	1762 (16.32%)	Ref.
	high-high	442 (4.09%)	1.01 (0.90, 1.13)
	low-low	3048 (28.22%)	0.99 (0.91, 1.08)
	high-low	5547 (51.37%)	1.03 (0.93, 1.13)
REOI			0.03 (-0.10, 0.15)
Mean of $PM_{2.5}$ (58.25 ug/m^3)			
P75 of NDVI (0.61)	low-high	1943 (17.99%)	Ref.
	high-high	261 (2.42%)	1.04 (0.90, 1.20)
	low-low	4146 (38.39%)	0.99 (0.91, 1.08)
	high-low	4449 (41.20%)	1.05 (0.95, 1.16)
REOI			0.02 (-0.14, 0.17)
P75 of $PM_{2.5}$ (71.00 ug/m^3)			
P75 of NDVI (0.61)	low-high	2093 (19.38%)	Ref.
	high-high	111 (1.03%)	0.98 (0.80, 1.21)
	low-low	5595 (51.81%)	1.00 (0.92, 1.08)
	high-low	3000 (27.78%)	1.07 (0.97, 1.19)
REOI			0.09 (-0.12, 0.30)

Note: * indicates the corresponding P-value < 0.05

Table S3 Excess mortality attributed to coexposure to exposure levels 4 by using $PM_{2.5}$ on lag01 as the exposure indicator.

Exposure levels of $PM_{2.5}$ and NDVI	Excess fraction	Number of excess deaths
$PM_{2.5}$≥35.00ug/m^3 and NDVI<0.30	4.86%	524.83
$PM_{2.5}$≥49.60ug/m^3 and NDVI<0.30	4.40%	475.16
$PM_{2.5}$≥58.25ug/m^3 and NDVI<0.30	4.31%	465.44
$PM_{2.5}$≥71.00ug/m^3 and NDVI<0.30	3.51%	379.04
$PM_{2.5}$≥35.00ug/m^3 and NDVI<0.43	2.08%	224.62
$PM_{2.5}$≥49.60ug/m^3 and NDVI<0.43	2.86%	308.85
$PM_{2.5}$≥58.25ug/m^3 and NDVI<0.43	3.33%	356.37
$PM_{2.5}$≥71.00ug/m^3 and NDVI<0.43	3.03%	327.21
$PM_{2.5}$≥35.00ug/m^3 and NDVI<0.61	0.00%	0.00
$PM_{2.5}$≥49.60ug/m^3 and NDVI<0.61	1.50%	161.99
$PM_{2.5}$≥58.25ug/m^3 and NDVI<0.61	1.96%	211.66
$PM_{2.5}$≥71.00ug/m^3 and NDVI<0.61	1.82%	196.54

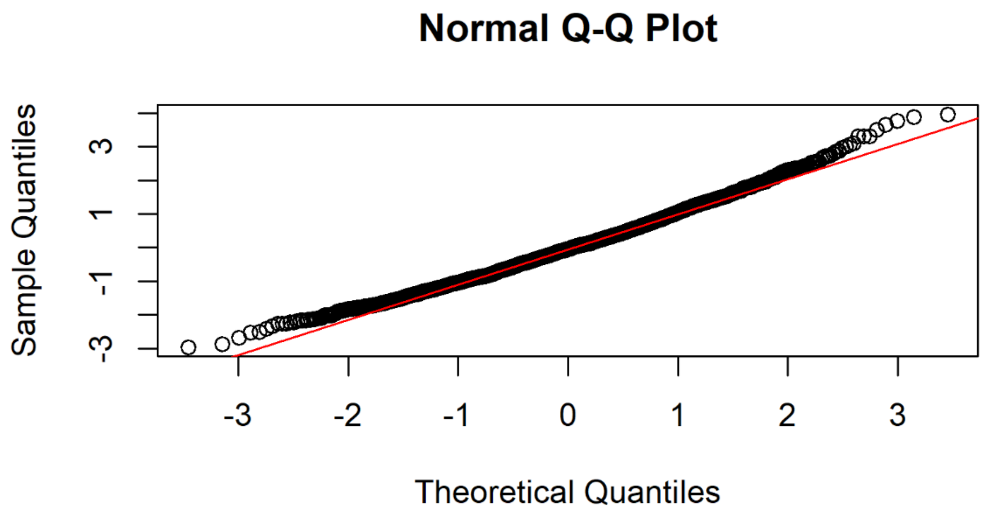

Figure S9. Normal Q-Q plot for main model.
